# Phylogeographic and evolutionary history analyses of the warty crab *Eriphia verrucosa* (Decapoda, Brachyura, Eriphiidae) unveil genetic imprints of a late Pleistocene vicariant event across the Gibraltar Strait, erased by postglacial expansion and admixture among refugial lineages

**DOI:** 10.1186/s12862-019-1423-2

**Published:** 2019-05-17

**Authors:** Temim Deli, Christina Kiel, Christoph D. Schubart

**Affiliations:** Zoology and Evolutionary Biology, University of Regensburg, D-93040 Regensburg, Germany

**Keywords:** Crustacea, Mitochondrial DNA, Mediterranean Sea, East Atlantic, Population genetics, Historical biogeography

## Abstract

**Background:**

The Pleistocene cyclic sea-level fluctuations are thought to have markedly affected the distribution and genetic architecture of Atlanto-Mediterranean biota. Despite the acknowledged key role played by these historical events in shaping population genetic structure of marine species, little is still known about the processes involved in shaping the spatial distribution of genetic variation within intertidal species. We intended in this study to reconstruct the phylogeography of a common and widely distributed coastal species across the East Atlantic and Mediterranean Sea (the warty crab *Eriphia verrucosa*), aiming to unravel potential microevolutionary processes likely involved in shaping its genetic polymorphism. For this purpose, a total of 155 specimens of *E. verrucosa* from 35 locations across the entire distribution range were analyzed by comparing a 453 basepairs region of the mitochondrial gene cytochrome oxidase subunit 1 (Cox1).

**Results:**

Our results unveiled the prevalence of high genetic connectivity among East Atlantic and Mediterranean populations, with noticeable genetic distinctiveness of the peripheral population from the Azores. Spatio-temporal patterns of genetic diversification and demographic history allowed retrieving genetic imprints of late Pleistocene vicariant event across the Gibraltar Strait followed by subsequent postglacial expansion events for both the East Atlantic and Mediterranean regions. Integrative evidences from the outcomes of comparison of regional genetic diversification, as well as evolutionary and biogeographic histories reconstructions, support the existence of potential glacial refugia for *E. verrucosa* in the East Atlantic and western Mediterranean. Our results also revealed low levels of genetic variability along with recent demographic and spatial expansion events for eastern Mediterranean warty crabs, suggesting that the eastern areas within the distribution range of the species might have been recently colonized from putative glacial refugia.

**Conclusions:**

These findings provide new insights into the phylogeography and evolutionary history of a common but poorly studied Atlanto-Mediterranean decapod species. Specifically, they contribute to the understanding of the impact of historical processes on shaping contemporary population genetic structure and diversity in intertidal marine species.

**Electronic supplementary material:**

The online version of this article (10.1186/s12862-019-1423-2) contains supplementary material, which is available to authorized users.

## Background

One of the main aims of evolutionary genetics is to discern the evolutionary processes responsible for driving and shaping geographic distribution of genetic variation of populations, as well as disentangling the origin of their genetic structure [1–4]. Integrative evidences from palaeogeographic, palaeoclimatic and phylogeographical investigations have brought in-depth knowledge to this issue, pointing out to the significant impact of Pleistocene climatic shifts on forging contemporary genetic polymorphisms [5–8].

The Pleistocene cyclic sea-level fluctuations are thought to have markedly affected the coastal environment and the evolutionary history of its biota [5] and regarded as one of the most important processes involved in shaping the contemporary geographic distribution of genetic variation [6, 9]. Noteworthy, these historic events have greatly influenced the distribution and evolutionary dynamics of populations [10, 11] following repeated habitat contractions and expansions of marine organisms. It has been postulated that habitat fragmentation induced by a lower sea level during glacial maxima could lead to a genetic bottleneck, with geographic isolates persisting in glacial refugia, and therefore to a heterogeneous population structure. Phylogeographic investigations across different parts of the globe have documented such patterns and allowed retrieving cryptic refugia in many marine species, such as in the seaweed *Palmaria palmata* across the English Channel [12] and the brown alga *Sargassum polycystum* across the southern Chinese coast [13]. The occurrence of phylogeographic breaks along with genetic imprints of glacial refugia have been also invoked in population genetic studies of East Atlantic and Mediterranean marine species such as in the gastropod *Nassarius nitidus* [14] and the green crab *Carcinus aestuarii* [8]. In contrast, range expansion primed by a rising sea level, following environmental warming during interglacials, could result in rapid population growth and consequent genetic homogeneity as a result of secondary contact between previously isolated evolutionary lineages [11]. The impact of these historical factors, intensified by the effects of contemporary environmental and oceanographic gradients, could have been potentially involved in shaping present day genetic variation and population structure in marine species [8, 15].

The Mediterranean Sea and the contiguous northEast Atlantic Ocean represent a suitable area to study biogeographical processes and investigate evolutionary patterns of diversification in marine species [2, 8, 16]. Indeed, the severe palaeogeographic and palaeoclimatic shifts that this region has undergone throughout its history, resulting in the onset of specific oceanographic features across its coastline, have set the stage for the impact of evolutionary and demographic processes on forging genetic variation of marine species [14, 17]. Recent phylogeographic investigations have provided evidence for the occurrence of pronounced genetic boundaries between the East Atlantic and the western Mediterranean, and between the Western and Eastern Mediterranean basins [2, 8, 15, 18–22]. Further pronounced genetic breaks have also been documented in the eastern Mediterranean, notably between the Adriatic-Ionian seas and the Aegean-Marmara-Black seas [1, 8, 22–26].

The above-mentioned biogeographic units could stem from the impact of historical, hydrographic and environmental processes. From a historical point of view, a marked drop of sea level during Pleistocene glaciation peaks [27] might have caused change in circulation patterns across main marine corridors, and consequently disrupted the biotic exchange across both the Gibraltar and Siculo-Tunisian Straits, leading to dramatic shifts in species range [19, 28, 29]. As a consequence, most of the current genetic patterns of marine coastal organisms could have been affected by past vicariance events, due to periodical closing of corridors that prevented larval interchange and gene flow [29]. This assumption has been consolidated by the outcome of recent phylogeographic investigations that have identified lower latitude Pleistocene marine refugia such as in the case of the seahorse *Hippocampus hippocampus* [30] and the intertidal gastropod *Nassarius nitidus* [14]. In addition to the potential impact of Quaternary climatic fluctuations (which might have left a strong footprint on the genetic structure of Atlanto-Mediterranean marine species), the contemporary oceanographic features (i.e., frontal systems and ocean currents) as well as gradual changes in abiotic factors (i.e., temperature and salinity) could also be involved in disrupting population connectivity [15, 18, 31]. Indeed, contemporary barriers to gene flow are mainly linked to the Almeria-Oran Oceanographic Front (accounting for the observed phylogeographic break between the East Atlantic and Mediterranean Sea) [19, 32] as well as to the particular hydrographic isolation patterns across the Siculo-Tunisian Strait [33] responsible for driving population genetic differentiation between Western and Eastern Mediterranean basins [4]. Besides, the potential interplay between oceanographic (involving mainly the isolating effects of Peloponnes anticyclonic front [34] and the Black Sea current) and environmental isolation of the Adriatic, Ionian and Aegean seas has been postulated to account for the genetic discontinuity recorded in the eastern Mediterranean [19, 23, 25].

Among Atlanto-Mediterranean marine biota, intertidal marine species are still less investigated in terms of their phylogeographic structure and evolutionary history. Furthermore, contemporary spatial distributions of their genetic diversity are thought to have been shaped by the impact of Pleistocene climate oscillations [35]. Hence, population genetic investigation of intertidal marine species would help not only with assessing the potential impact of postulated biogeographic boundaries within the East Atlantic and Mediterranean, but also unraveling the effect of microevolutionary processes (including glacial-induced fragmentation and postglacial-induced recolonisation) on forging patterns of genetic variability and structure.

The warty crab *Eriphia verrucosa* (Forskål, 1775) (Decapoda, Brachyura, Eriphiidae) represents a good model to address the above mentioned issues. This littoral decapod is one of the most ecologically important coastal species, playing a crucial role in structuring intertidal communities, as a highly predatory shore crab [36, 37]. It has a wide geographic distribution, stretching from the Mediterranean Sea (including the Black Sea) to the East Atlantic Ocean from Brittany to Mauritania and the Azores [38, 39]. The species inhabits stony coastal zones and occupies infralittoral crevices at shadow zones with a well-developed algal coverage. It mainly occurs in the lower intertidal zone (being more abundant in the infra and mesolittoral zones than in the supralittoral) [39] where it can be encountered among stones and seaweeds along rocky coastlines in shallow waters down to depths of 15 m [40]. The reproduction of *E. verrucosa* begins in May or June [41]. Spawning occurs from late July to the end of August [42], depending on water temperature. The warty crab is considered as very prolific, being characterized by high fecundity [41–43]. The dispersal potential of the species is probably high, since the complete larval development takes place in the ocean with four zoeal stages and one megalopa stage [44]. According to the experimental study held by Lumare and Gozzo [44], larval development of *E. verrucosa* can take 49 days at a temperature of 21 °C. Considering all of these eco-biological aspects, genetic homogeneity and panmixia could be expected among populations of this crustacean species. However, recent phylogeographic investigations, carried out in other Atlanto-Mediterranean decapod species (with similar life-history traits as *E. verrucosa*), revealed restricted patterns of gene flow, such as in the green crab *Carcinus aestuarii* [4, 8], the marbled crab *Pachygrapsus marmoratus* [21] and the littoral prawn *Palaemon elegans* [2, 15]. The outcome of these studies pointed out to the involvement of other factors, such historical isolating processes, contemporary oceanographic discontinuities as well as larval behavior, in shaping patterns of population genetic structure of littoral decapods.

**Fig. 1:**
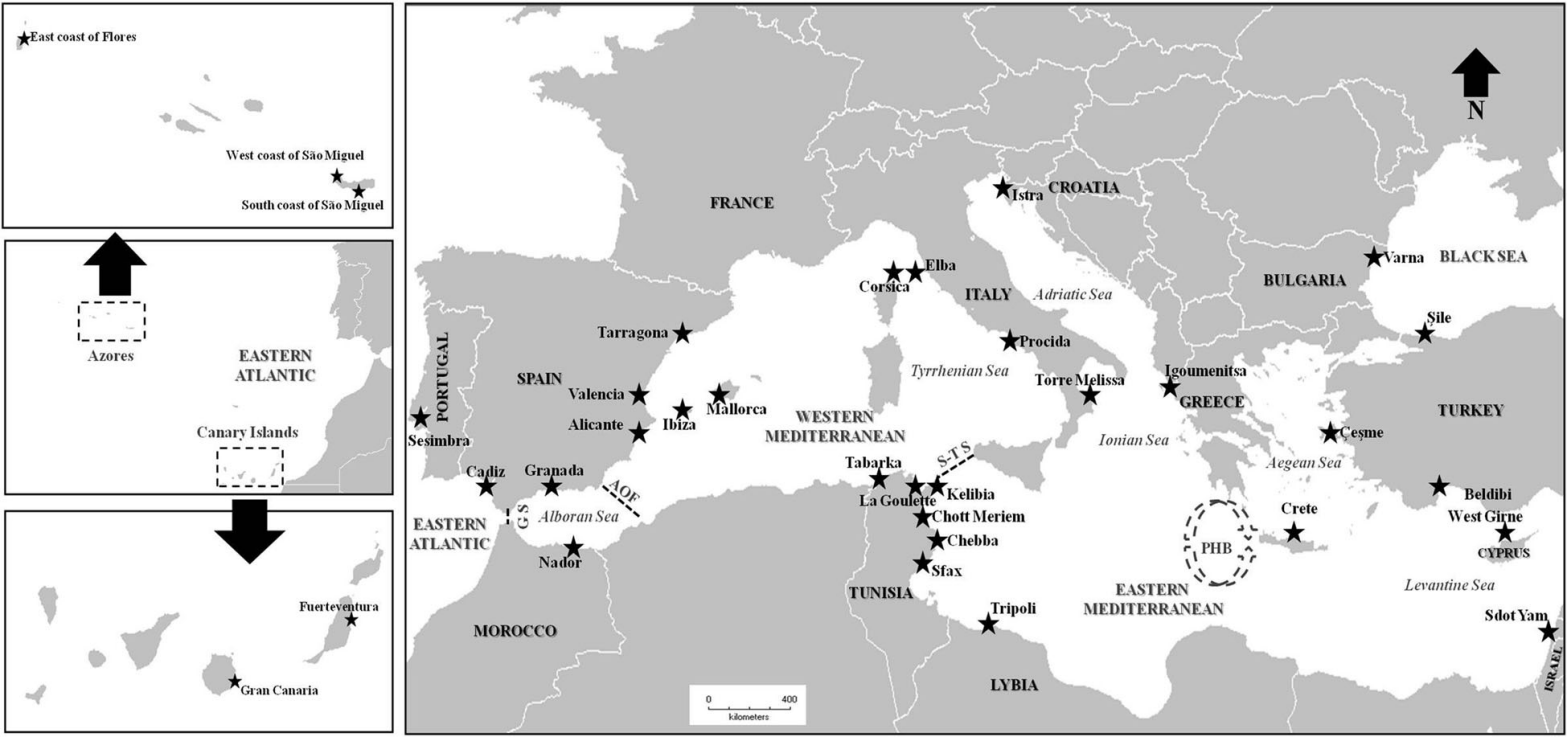
Sampling locations of *Eriphia verrucosa* across the East Atlantic and Mediterranean Sea. The main phylogeographic breaks are shown as dotted lines. G S: Gibraltar Strait; AOF: Almería-Oran Oceanographic Front; S-T S: Siculo-Tunisian Strait; PHB: Peloponnese Hydrographic Break (represented by the quasi-circular anti-cyclonic feature southwest of Peloponnese). The base map was constructed with the software DIVA-GIS 7.5.0 (http://www.diva-gis.org)

In light of these considerations, in this study we investigated the following questions: (1) Can the main postulated barriers to gene flow spanning the East Atlantic and the Mediterranean Sea restrict gene flow among populations of *E. verrucosa* allowing for the occurrence of significant patterning of population genetic structure? (2) Did palaeoclimatic and palaeogeographical evolution of the surveyed geographic area during the Quaternary significantly impact contemporary spatial distribution of genetic polymorphisms and their variations through space and time, and what are the potential evolutionary processes likely involved in shaping genetic diversity and affecting spatial genetic structure within this decapod species? In order to answer these questions, we examined the mitochondrial phylogeography of the species and reconstruct its evolutionary and demographic histories. An intensive sampling of *E. verrucosa* was conducted from its entire distribution range, stretching from the East Atlantic to the Black Sea (Fig. 1 and Table 1), and sequences of the mitochondrial cytochrome oxidase subunit 1 (Cox1) gene were generated and compared. When amplifying this marker in the sampled specimens, we made sure that we sampled the region of the gene that has been shown to be variable enough for unveiling significant genetic differentiation in previous studies on marine decapods [8, 15, 16]. Furthermore, the use of this marker allowed us to incorporate all previously published Cox1 sequences into the analyses in order to increase the size of the examined dataset. However, given that only small portion of the mtDNA genome was analyzed, we combined several analytical tools in order to recover the maximum amount of information contained in the mtDNA at different hierarchical levels (genealogy, phylogeographic structure, calibrated phylogeny, ancestral area reconstruction, and historical demography). Although the exclusive use of uniparentally inherited markers can provide a distorted understanding of phylogeographic structure, the smaller effective population size of mtDNA as compared to nuclear loci makes mtDNA an appropriate molecular marker for reconstructing species evolutionary histories [45].

**Table 1:** Sampling information on East Atlantic and Mediterranean specimens of *Eriphia verrucosa* including collection sites, countries, regions, and geographic coordinates. N: the examined number of specimens for each population

Collection site	Country	Region	Geographic coordinates	N
Azores: West coast of São Miguel	Portugal	East Atlantic	37°51'41"N 25°51'17"W	10
Azores: South coast of São Miguel (*a)	Portugal	East Atlantic	39°27'36"N 31°07'48"W	4
Azores: East coast of Flores (*b)	Portugal	East Atlantic	37°42'00"N 25°30'00"W	4
Canary Islands: East coast Gran Canaria	Spain	East Atlantic	27°50'00"N 15°25'14"W	10
Canary Islands: Fuerteventura, Matorral	Spain	East Atlantic	28°25'30"N 13°51'26"W	7
Setúbal: Sesimbra	Portugal	East Atlantic	38°26'28"N 09°6'35.50"W	6
Cádiz: Corrales de Rota (*c)	Spain	East Atlantic	36°31'47"N 06°17'40"W	1
Nador Lagoon	Morocco	Alboran Sea	35°12'16"N 02°54'47"W	2
Granada: Almuñecar	Spain	Alboran Sea	36°43'44"N 03°41'37"W	1
Alicante: Moraira, El Portet	Spain	western Mediterranean	38°41'11"N 00°08'51"E	1
Alicante: Moraira, Playa de l’Ampolla	Spain	western Mediterranean	38°41'12"N 00°07'53"E	9
Valencia: Puebla de Farnals	Spain	western Mediterranean	39°34'14"N 00°16'34"W	4
Ibiza: Cala Llenya	Spain	western Mediterranean	39°00'58"N 01°35'09"E	3
Mallorca, Portals Vells	Spain	western Mediterranean	39°28'26"N 02°31'16"E	1
Tarragona: L’Ampolla	Spain	western Mediterranean	40°48'57"N 0°43'18"E	11
Corsica: Calvi: Stareso	France	western Mediterranean	42°34'49"N 08°43'27"E	6
Elba: Capoliveri	Italy	western Mediterranean	42°44'54"N 10°21'33"E	3
Procida: Procida harbour	Italy	western Mediterranean	40°45'57"N 14°02'02"E	1
Tabarka	Tunisia	western Mediterranean	36°57'27"N 08°45'05"E	1
La Goulette	Tunisia	western Mediterranean	36°49'03"N 10°18'19"E	1
Kelibia	Tunisia	western Mediterranean	36°51'00"N 11°06'00"E	1
Chott Meriem	Tunisia	eastern Mediterranean	35°54'54"N 10°33'37"E	9
Chebba	Tunisia	eastern Mediterranean	35°13'20"N 11°03'36"E	1
Sfax	Tunisia	eastern Mediterranean	34°44'43"N 10°45'41"E	2
Tripoli	Lybia	eastern Mediterranean	32°52'34"N 13°11'15"E	7
Istra: Pula: Valsaline	Croatia	eastern Mediterranean	44°51'01"N 13°50'01"E	16
Calabria: Torre Melissa	Italy	eastern Mediterranean	39°17'59"N 17°06'35"E	3
Igoumenitsa: Kalami	Greece	eastern Mediterranean	39°28'23"N 20°14'22"E	5
Crete: Iraklion	Greece	eastern Mediterranean	35°20'40"N 25°08'10"E	6
West of Çesme	Turkey	eastern Mediterranean	38°19'43"N 26°17'44.5"E	1
Lykia: Beldibi	Turkey	eastern Mediterranean	36°41'54"N 30°34'23"E	3
West of Girne	Cyprus	eastern Mediterranean	35°20'34"N 33°18'05"E	10
Sdot Yam	Israel	eastern Mediterranean	32°29'28"N 34°53'08"E	1
Varna	Bulgaria	Black Sea	43°12'15"N 27°55'59"E	1
Sile (*d)	Turkey	Black Sea	41°10'35"N 29°36'14"E	3

*: Cox1 sequences retrieved from GenBank. Accession numbers for (a): JQ306070, JQ306072, JQ306076, JQ306077. Accession numbers for (b): JQ306071, JQ306074, JQ306075, JQ306078. Accession numbers for (c): HM638038. Accession numbers for (d): KP136637, KP136680, KP136681

## Results

### Sequence variation, genetic diversity and assessment of regional genetic diversification

**Fig. 2:**
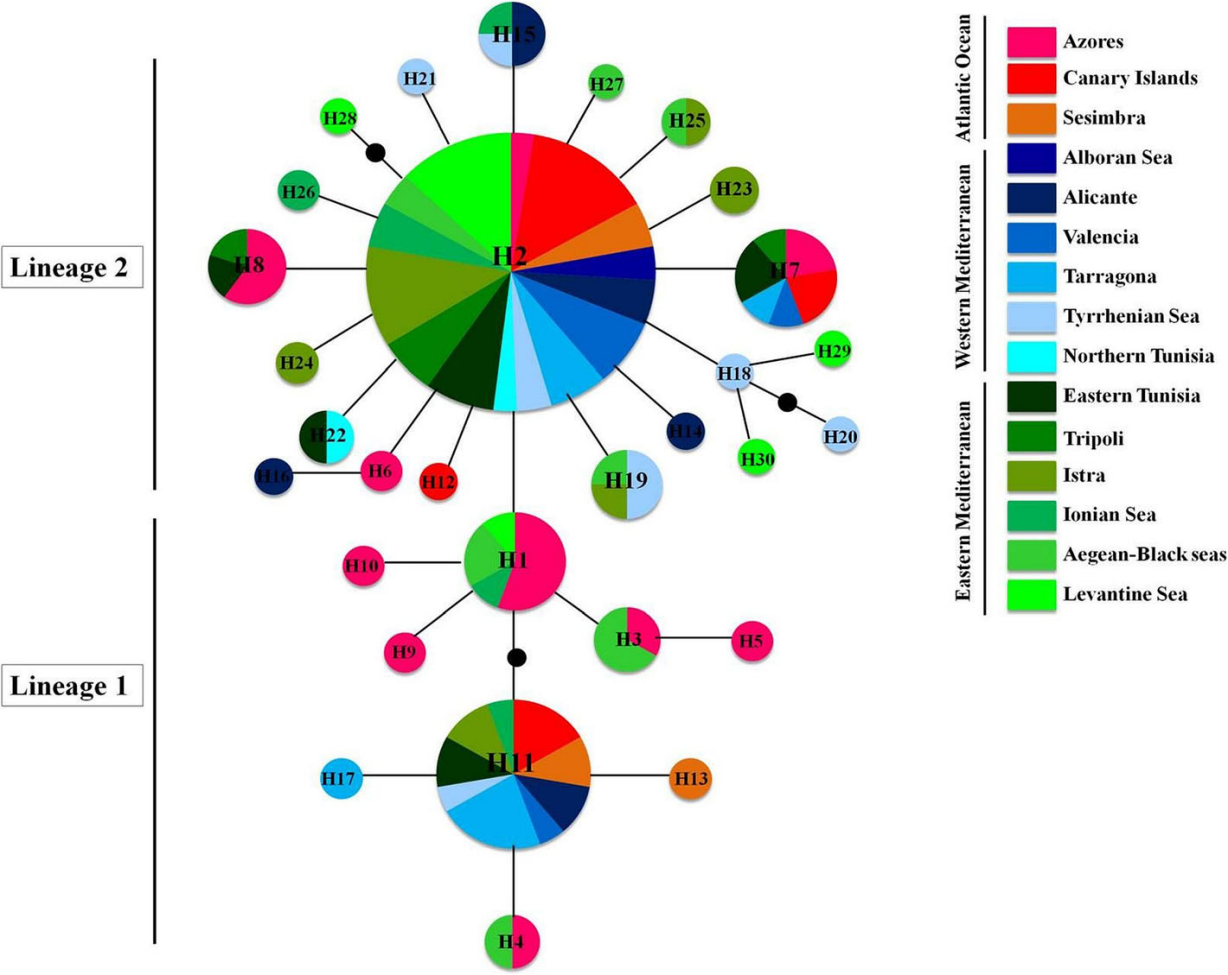
TCS parsimony network of *Eriphia verrucosa*, based on the alignment of 453 bp of the mitochondrial gene Cox1, showing the evolutionary relationships among the recorded haplotypes. Haplotype 2 corresponds to the ancestral haplotype. Small black circles correspond to missing (or hypothetical) haplotypes. Each connecting line indicates one mutational step between haplotypes. Circle sizes depict proportions of haplotypes; the smallest corresponds to 1 and the largest to 77 individuals. Cox1 lineages of *E. verrucosa* were defined based on the outcomes of both BEAST and RASP analyses (see Figs. 3 and 4)

A total of 30 Cox1 haplotypes were identified in the 155 sampled individuals of *E. verrucosa* (Fig. 2 and Table 2). The newly generated sequences (147) allowed detecting 27 haplotypes; while the 12 sequences retrieved from GenBank contributed to the haplotype dataset by only 3 haplotypes (Additional file 1: Table S1). Details on the pattern of assignment of these latter sequences to the detected haplotypes in this study are given in Additional file 2: Table S2. The difference among the obtained haplotypes was due to 29 variable sites of which 12 were parsimony-informative and 17 were autapomorphic. The outcome of MEGA analysis retrieved unbalanced nucleotide composition of the analyzed Cox1 fragment (C = 20.46%; T = 34.96%; A = 27.36%; G = 17.22%), showing an A-T bias as expected for invertebrate mitochondrial DNA [46].

**Table 2:** Genetic diversity and historical demographic results for examined specimens of *Eriphia verrucosa*. Values reported for each population, each region (and sub-region), as well as for the total dataset are: Sample size (*N*), number of haplotypes (*N*h), number of polymorphic sites (*N*ps), haplotype diversity (*h*), nucleotide diversity (*π*), mean number of nucleotide differences (K), Tajima’s *D* test (*D*), Fu’s *F*_*S*_ test (*F*_*S*_), Ramos-Onsins and Rozas’s *R*_2_ test (*R*_2_), and mismatch distribution raggedness index (*rg*)

	*N*	*N*h	*N*ps	*h*	*π*	K	*D*	*F*_*S*_	*R*_*2*_	*rg*
Population/Region/*Sub-region*										
Azores	18	10	10	0.901 ± 0.050	0.0043 ± 0.0006	1.986	-1.140	**-5.133**	**0.087**	0.085
Canary Islands	17	4	5	0.566 ± 0.123	0.0027 ± 0.0008	1.264	-0.467	0.349	0.124	0.175
Sesimbra	7	3	4	0.666 ± 0.160	0.0044 ± 0.0010	2.000	1.075	1.320	0.231	0.283
Atlantic Ocean	42	13	12	0.808 ± 0.052	0.0039 ± 0.0005	1.786	-1.097	**-6.147**	0.069	0.041
Alboran Sea	3	1	0	0.000 ± 0.000	0.0000 ± 0.0000	0.000	0.000	0.000	N.D	**0.000**
Alicante	10	5	7	0.822 ± 0.097	0.0044 ± 0.0010	2.022	-0.774	-0.577	0.136	0.060
Valencia	8	3	4	0.464 ± 0.200	0.0022 ± 0.0012	1.000	**-1.534**	0.204	0.250	0.228
Tarragona	11	4	5	0.709 ± 0.099	0.0044 ± 0.0006	2.000	0.657	0.768	0.180	0.236
Tyrrhenian Sea	10	7	8	0.911 ± 0.077	0.0044 ± 0.0010	2.022	-1.229	**-3.237**	**0.112**	0.107
Northern Tunisia	3	2	1	0.666 ± 0.314	0.0014 ± 0.0006	0.666	0.000	0.200	0.471	0.555
*western Mediterranean*	45	12	14	0.711 ± 0.065	0.0037 ± 0.0005	1.688	**-1.459**	**-5.038**	**0.058**	0.069
Eastern Tunisia	12	5	6	0.742 ± 0.116	0.0034 ± 0.0009	1.545	-0.847	-0.831	**0.118**	0.123
Tripoli	7	3	2	0.523 ± 0.209	0.0012 ± 0.0005	0.571	-1.237	-0.921	0.225	0.185
Istra	16	6	6	0.683 ± 0.120	0.0028 ± 0.0007	1.275	-1.009	-1.943	**0.101**	0.098
Ionian Sea	8	5	5	0.785 ± 0.151	0.0031 ± 0.0010	1.428	-1.175	-1.916	**0.147**	0.137
Aegean-Black seas	11	7	6	0.909 ± 0.066	0.0040 ± 0.0008	1.854	-0.374	**-3.120**	0.131	0.100
Levantine Sea	14	5	6	0.505 ± 0.158	0.0021 ± 0.0008	0.978	**-1.728**	-1.690	**0.104**	0.296
*eastern Mediterranean*	68	18	18	0.697 ± 0.062	0.0029 ± 0.0004	1.336	**-1.935**	**-14.273**	**0.036**	0.035
Mediterranean Sea	113	24	25	0.702 ± 0.046	0.0032 ± 0.0003	1.483	**-2.014**	**-20.266**	**0.029**	0.037
Total	155	30	29	0.733 ± 0.036	0.0034 ± 0.0002	1.575	**-2.008**	**-27.328**	**0.026**	0.034

Significant values in bold. Non significant values for the raggedness index (*rg*) accepting the null hypothesis of expectation under a sudden demographic expansion model. N.D: Given the lack of polymorphism in the Alboran Sea, the *R*_2_ index cannot be computed. Azores (west and south coasts of São Miguel, and east coast of Flores), Canary Islands (Gran Canaria and Fuerteventura), Sesimbra (Sesimbra and Cadiz), Alboran Sea (Nador and Granada), Alicante (Moraira, including El Portet), Valencia (Valencia, Ibiza, and Mallorca), Tarragona, Tyrrhenian Sea (Corsica, Elba, and Procida), Northern Tunisia (Tabarka, La Goulette, and Kelibia), Eastern Tunisia (Chott Meriem, Chebba, and Sfax), Tripoli, Istra, Ionian Sea (Torre Melissa and Igoumenitsa), Aegean-Black seas (Crete, Çesme, Sile, and Varna), and Levantine Sea (Beldibi, West Girne, and Sdot Yam)

An analysis of genetic diversity of the examined Cox1 dataset showed high total haplotype diversity (*h* = 0.733 ± 0.036) and low total nucleotide diversity (*π* = 0.0034 ± 0.0002). The total mean number of nucleotide differences (K) was 1.575. Details on these parameters of genetic variability in each examined population are reported in Table 2. At the regional scale, the East Atlantic specimens of *E. verrucosa* were shown to be genetically more variable than their Mediterranean counterparts (Table 2). Within the Mediterranean Sea, warty crabs from the Western Basin exhibited higher levels of genetic diversity than those from the Eastern Basin (Table 2). As nine out of the twelve GenBank sequences derived from the East Atlantic (eight from the Azores and one from Cádiz, Table 1), the recorded higher levels of genetic diversity in the East Atlantic than those observed in the Mediterranean could have been overestimated due to the inclusion of those sequences in the analysis. However, when re-analyzing the levels of three genetic diversity parameters (*h*, *π*, and K) in a dataset excluding GenBank sequences, we noticed that East Atlantic specimens of *E. verrucosa* remain genetically more diversified than their Mediterranean counterparts (Additional file 1: Table S1). This indicates that inclusion of GenBank sequences has no influence on the outcome of our genetic diversity analyses.

Assessment and statistical comparison of seven genetic diversification parameters among three examined regions (Atlantic, western Mediterranean and eastern Mediterranean), after a rarefaction procedure involving 30 replicates, showed that the East Atlantic and western Mediterranean samples were significantly more diversified than their eastern Mediterranean counterparts for all parameters (except for haplotypic richness) (Table 3). Significant differences between the East Atlantic and western Mediterranean were also unveiled for all genetic diversity indices, with the former region being more variable than the latter (Table 3). Increase in the number of replicates did not influence the outcome of inter-regional comparison, as the same results were retrieved when re-carrying out the analyses with 60 replicates (Table 3).

**Table 3:** Assessment and comparison of genetic diversification levels among the three examined regions (Atlantic (A), western Mediterranean (WM) and eastern Mediterranean (EM)) for *Eriphia verrucosa*. The computed values for each parameter were obtained after a rarefaction procedure (as described in the Methods section) to the smallest sample size (*N*) of 42 (from the Atlantic)

Genetic diversity indices	Number of replicates (R)	A (42)	WM (42)	EM (42)	Assessment of inter-regional difference (unpaired *t*-test)		
					A vs. WM	A vs. EM	WM vs. EM
Haplotype diversity (*h*)	R = 30	0.808	0.728	0.677	***	***	***
	R = 60	0.808	0.728	0.667	***	***	***
Nucleotide diversity (*π*)	R = 30	0.0039	0.0038	0.0028	**	***	***
	R = 60	0.0039	0.0038	0.0027	***	***	***
Mean number of nucleotide differences (K)	R = 30	1.786	1.748	1.272	**	***	***
	R = 60	1.786	1.744	1.255	***	***	***
Haplotypic richness (*Hap*r)	R = 30	13	11.800	12.200	***	*	ns
	R = 60	13	11.800	12.216	***	***	ns
Number of private haplotypes (*N*p)	R = 30	7.500	6.400	4.466	***	***	***
	R = 60	7.566	6.550	4.716	***	***	***
Proportion of private haplotypes (*N*p/*N*)	R = 30	0.178	0.152	0.106	***	***	***
	R = 60	0.180	0.155	0.112	***	***	***
Genetic endemism (*N*p/*Hap*r)	R = 30	0.576	0.542	0.361	*	***	***
	R = 60	0.582	0.554	0.381	*	***	***

*: Significant difference at *P* < 0.05; **: Significant difference at *P* < 0.01; ***: Significant difference at *P* < 0.001; *ns* Non-significant difference (*P* > 0.05). The values, highlighted for each diversity measure, correspond to the mean values obtained for the examined number of replicates. The number of private haplotypes, for each replicate, was determined according to the pattern of distribution of haplotypes in a dataset composed of 126 samples taken randomly from the East Atlantic (42), western Mediterranean (42) and the eastern Mediterranean (42)

### Phylogenetic relationships among Cox1 haplotypes

**Fig. 3:**
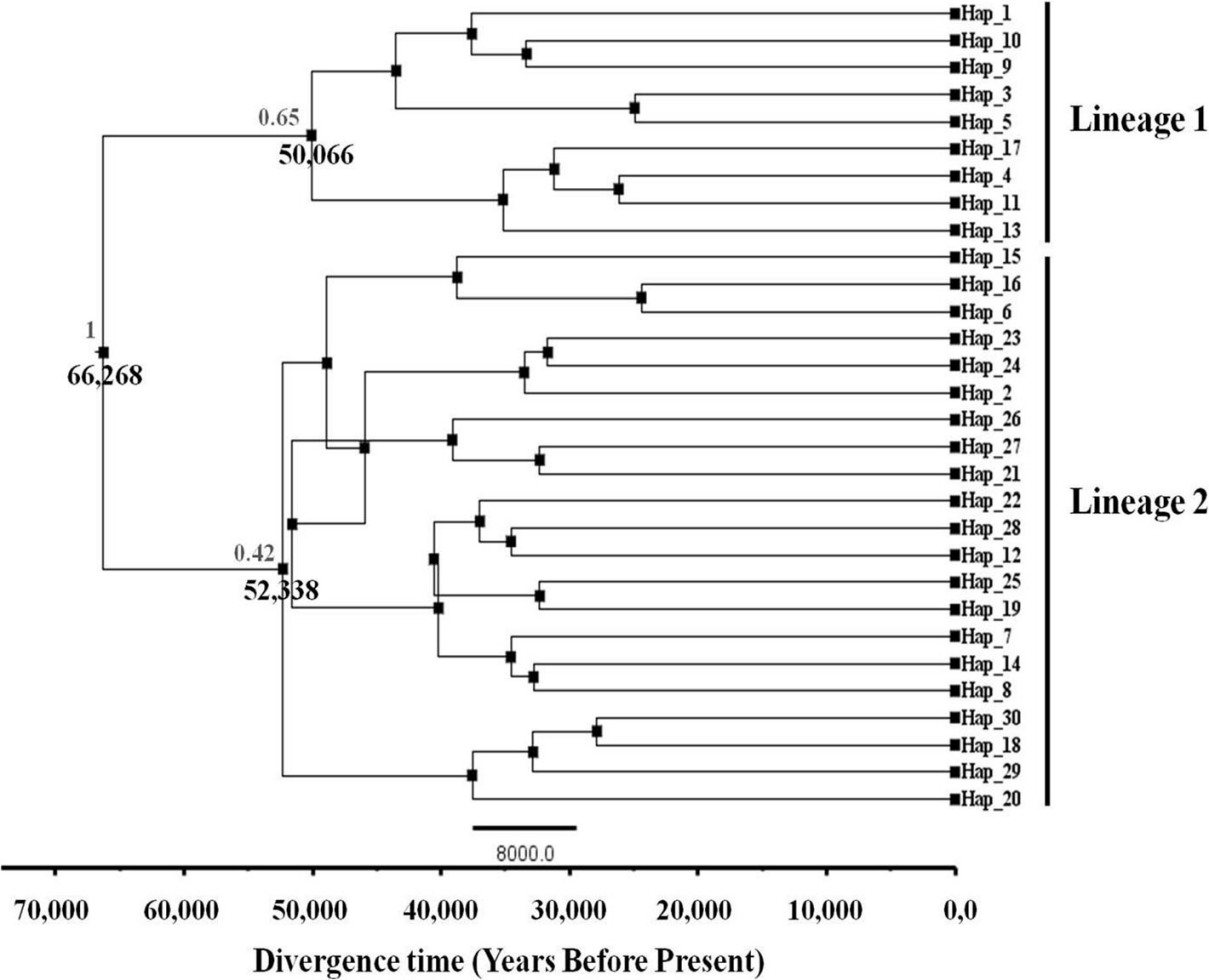
Bayesian phylogenetic analysis (as implemented in BEAST) exhibiting diversification pattern of the retrieved Cox1 haplotypes wihin *Eriphia verrucosa* through time. Node ages (mean values in black below the nodes) are highlighted in years before present (YBP) for the three main nodes. Values in grey (above the main nodes) correspond to the posterior probabilities (Bayesian inference) of the generated nodes. The specifically estimated Cox1 gene mutation rate, for *Eriphia*, of 4.87% per Myr was used to calibrate the genealogy and date tMRCA of Cox1 lineages

**Fig. 4:**
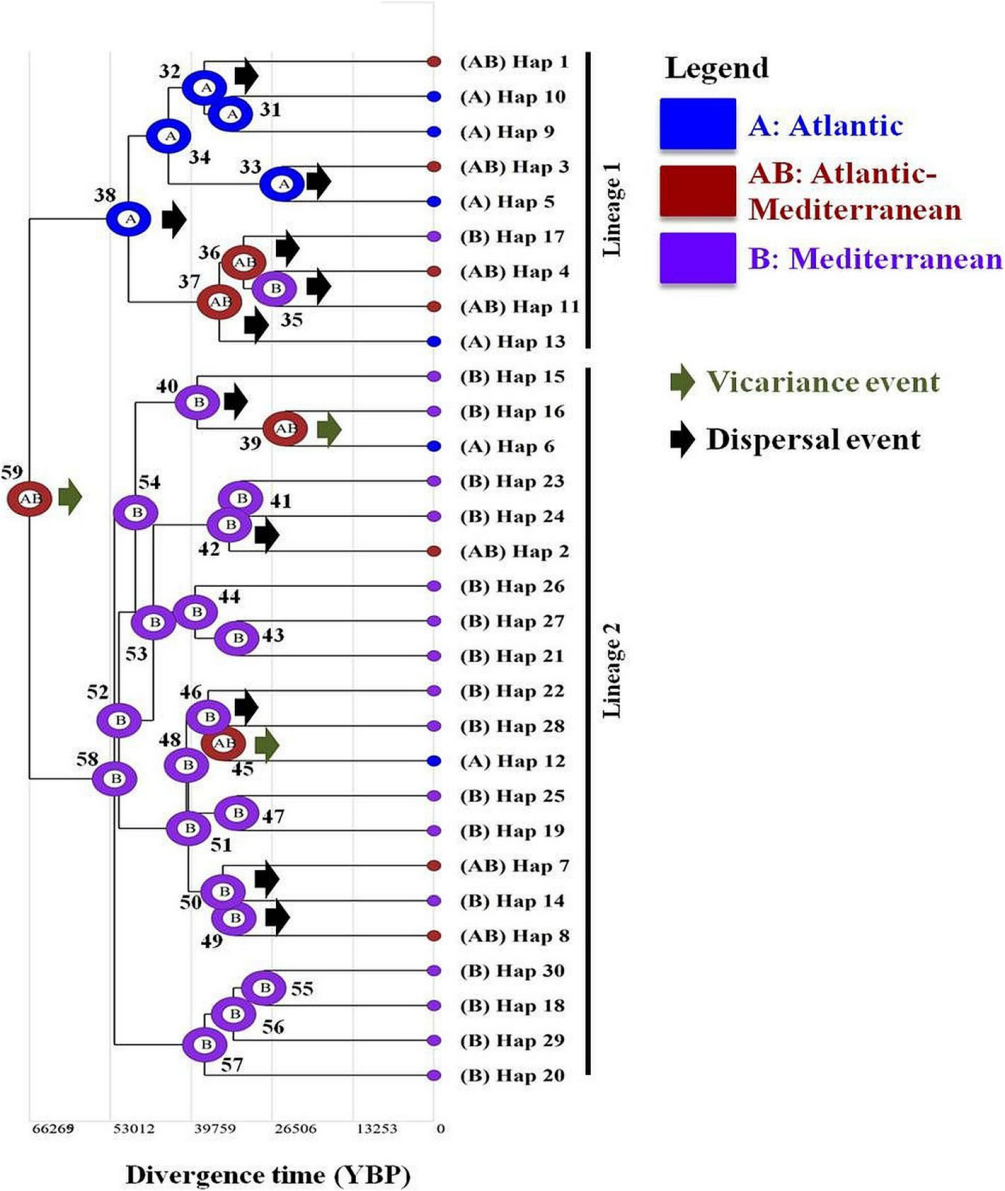
Biogeographic history of *Eriphia verrucosa*, highlighting ancestral area reconstruction based on Cox1 haplotypes. Couloured circles on each node (next to corresponding number) show the likelihood of occurrence of each ancestral haplotype at an inferred ancestral biogeographic region. Graphical results of ancestral distributions at each node of the phylogeny were obtained by S-DIVA (Statistical Dispersal-Vicariance Analysis) method, as implemented in RASP version 3.2. Biogeographic regions are shown in different colors and denoted with alphabetic letters (A, B, and AB). Green arrows indicate vicariance events at the corresponding nodes; while black arrows highlight possible dispersal events

The TCS statistical parsimony procedure yielded two resolved sub-networks centered around two main and common haplotypes (haplotype 2 and haplotype 11) (Fig. 2). These two clades or lineages were defined and delineated according to the outcome of the Bayesian phylogenetic analyses (Figs. 3 and 4). Both retrieved Cox1 lineages were separated by one mutational step. The two common haplotyes 11 (within lineage 1) and 2 (within lineage 2), separated by three mutational steps, were found in the East Atlantic as well as in the Western and Eastern Mediterranean basins (Fig. 2 and Additional file 3: Table S3). Such finding, showing no significant phylogeographic patterning, was supported by the outcome of PERMUT analysis. Indeed, the *N*_ST_ value (0.061) was not significantly higher than the *G*_ST_ value (0.048); *P* > 0.05, highlighting lack of a significant relationship between genealogy and geographic distribution of Cox1 haplotypes. Within lineage 1, a further separation by two mutational steps was noticed between two sets of haplotypes (or sub-clades), centered around haplotypes 1 and 11. The sub-clade around haplotype 1 was shown to be slightly prevailing in the East Atlantic. However, such observation should be interpreted with caution as this sub-clade is relatively common in a small subset of specimens from the Azores. Besides, the central haplotype 1 and the derived haplotype 3 have been also found in the eastern Mediterranean and may occur in the western Mediterranean but they probably went unnoticed because of the small sample size obtained for this region (only 45 specimens).

### Patterns of genetic differentiation and phylogeographic structure

Genetic differentiation among studied populations of *E. verrucosa* was found to be weak but significant based on both Tajima-Nei distances (*Φ*_ST_ = 0.043, df = 154, *P* = 0.026) and haplotype frequencies (*F*_ST_ = 0.045, df = 154, *P* = 0.008). Pairwise comparisons of genetic differentiation showed that only five (based on nucleotide divergence) and six (based on haplotype frequencies) out of the 105 pairwise genetic distances were significant after the B-Y FDR correction (Table 4). Most of the significant genetic distances, computed from nucleotide divergence and haplotype frequencies, were due to the genetic distinctiveness of the population of the Azores (Table 4). Re-analysis of pairwise genetic differentiation in a total dataset of 143 specimens (excluding the twelve sequences from GenBank) also unveiled marked genetic differentiation of the Azores population (Additional file 4: Table S4).

**Table 4:** Pairwise comparisons of genetic differentiation, in *Eriphia verrucosa*, estimated from nucleotide divergence (*Φ*_ST_, below the diagonal) and haplotype frequency (*F*_ST,_ above the diagonal). Significant values in boldface (*P* < 0.05) were calculated from 10,000 permutations. These significance values were subjected to a B-Y FDR correction [85], rendering a critical value of *P* < 0.00954; values that remain significant after the correction are marked with asterisks

	Atlantic Ocean			western Mediterranean						eastern Mediterranean					
	AZO	CAI	SES	ALB	ALC	VAL	TAR	TYR	NTU	ETU	TRI	IST	ION	AG-B S	LEV
AZO	§	0.196*	0.144*	**0.274**	**0.094**	0.208*	0.135*	**0.062**	0.108	**0.094**	**0.158**	0.152*	0.066	−0.001	0.209*
CAI	**0.083**	§	−0.057	−0.024	0.025	−0.082	−0.006	0.076	−0.061	−0.040	− 0.048	−0.017	−0.022	**0.116**	0.002
SES	0.090	0.052	§	0.045	− 0.046	− 0.052	− 0.082	0.006	− 0.076	− 0.059	− 0.005	− 0.050	− 0.071	0.057	0.020
ALB	0.025	− 0.108	0.125	§	0.111	−0.110	0.142	0.147	0.000	0.043	−0.089	−0.003	0.025	0.168	−0.094
ALC	0.060	−0.022	0.001	−0.114	§	0.038	−0.026	−0.031	− 0.040	−0.019	0.046	−0.000	− 0.072	0.027	0.079
VAL	0.060	−0.089	0.074	−0.175	−0.050	§	0.018	0.087	−0.090	−0.042	− 0.105	−0.031	− 0.025	**0.121**	− 0.046
TAR	**0.115**	0.077	−0.108	0.144	0.042	0.093	§	0.022	0.004	−0.041	0.059	0.004	−0.025	0.076	0.105
TYR	0.116*	0.001	0.043	−0.114	− 0.031	−0.033	0.082	§	−0.023	0.009	0.072	0.017	−0.030	−0.011	**0.110**
NTU	0.073	−0.011	0.147	0.000	−0.049	−0.043	0.172	−0.050	§	−0.115	− 0.111	−0.083	− 0.106	−0.001	− 0.081
ETU	0.055	−0.060	0.042	−0.128	−0.027	− 0.097	0.065	− 0.007	−0.087	§	−0.051	− 0.020	−0.046	0.044	0.032
TRI	0.107	0.008	0.251	−0.166	0.036	−0.051	0.247	0.036	0.017	−0.035	§	−0.019	−0.022	0.092	−0.049
IST	0.114*	−0.025	0.083	−0.134	−0.014	− 0.061	0.118	− 0.005	−0.031	− 0.023	0.018	§	−0.040	0.051	0.004
ION	0.019	−0.049	0.024	−0.132	−0.082	− 0.079	0.062	− 0.034	−0.043	− 0.051	0.026	− 0.039	§	− 0.010	−0.006
AG-B S	−0.018	0.051	0.032	0.006	0.026	0.036	0.067	0.061	0.056	0.041	0.137	0.060	−0.007	§	**0.115**
LEV	0.145*	0.048	**0.244**	−0.174	0.058	−0.005	0.261*	0.030	−0.035	0.040	−0.004	0.035	0.020	0.134*	§

*AZO* Azores, *CAI* Canary Islands, *SES* Sesimbra, *ALB* Alboran Sea, *ALC* Alicante, *VAL* Valencia, *TAR* Tarragona, *TYR* Tyrrhenian Sea, *NTU* Northern Tunisia, *ETU* Eastern Tunisia, *TRI* Tripoli, *IST* Istra, *ION* Ionian Sea, *AG-B S* Aegean-Black seas, *LEV* Levantine Sea

Examination of phylogeographic structure across potential barriers to gene flow, encompassing the East Atlantic and Mediterranean Sea, revealed none significant genetic subdivision within *E. verrucosa* across the Gibraltar Strait, the Almería-Oran Oceanographic Front, the Siculo-Tunisian Stait, and the Peloponnese hydrographic break (Table 5). The lack of genetic structure across these biogeographic boundaries was recorded according to both nucleotide divergence (Tajima and Nei distance) and haplotype frequencies (Table 5).

**Table 5:** Analysis of molecular variance (AMOVA) testing for partition of the genetic variance among populations of *Eriphia verrucosa* under different biogeographic hypotheses

Tested hypotheses of population genetic structure across potential barriers to gene flow	F-statistics based on Tajima and Nei distance	F-statistics based on haplotype frequency
1-Genetic structure across the Gibraltar Strait:		
Atlantic Ocean (Azores, Canary Islands, Sesimbra) vs. Mediterranean Sea (Alboran Sea, Alicante, Valencia, Tarragona, Tyrrhenian Sea, Northern Tunisia, Eastern Tunisia, Tripoli, Istra, Ionian Sea, Aegean-Black seas, Levantine Sea)	*Φ*_SC_ = 0.040 * *Φ*_ST_ = 0.048 * *Φ*_CT_ = 0.008 ns	_FSC_ = 0.045 ** _FST_ = 0.044 ** _FCT_ = − 0.001 ns
2-Genetic structure across the Almería-Oran Oceanographic Front:		
Atlantic Ocean-Alboran Sea (Azores, Canary Islands, Sesimbra, Alboran Sea) vs. Mediterranean Sea (Alicante, Valencia, Tarragona, Tyrrhenian Sea, Northern Tunisia, Eastern Tunisia, Tripoli, Istra, Ionian Sea, Aegean-Black seas, Levantine Sea)	*Φ*_SC_ = 0.043 * *Φ*_ST_ = 0.044 * *Φ*_CT_ = 0.000 ns	_FSC_ = 0.049 ** _FST_ = 0.039 ** _FCT_ = − 0.010 ns
3-Genetic structure across the Siculo-Tunisian Strait:		
Atlantic Ocean-western Mediterranean (Azores, Canary Islands, Sesimbra, Alboran Sea, Alicante, Valencia, Tarragona, Tyrrhenian Sea, Northern Tunisia) vs. eastern Mediterranean (Eastern Tunisia, Tripoli, Istra, Ionian Sea, Aegean-Black seas, Levantine Sea)	*Φ*_SC_ = 0.041 * *Φ*_ST_ = 0.045 * *Φ*_CT_ = 0.003 ns	_FSC_ = 0.048 ** _FST_ = 0.041 ** _FCT_ = − 0.007 ns
4-Genetic structure across the Peloponnese hydrographic break:		
Atlantic Ocean-western Mediterranean-eastern Mediterranean (Azores, Canary Islands, Sesimbra, Alboran Sea, Alicante, Valencia, Tarragona, Tyrrhenian Sea, Northern Tunisia, Eastern Tunisia, Tripoli, Istra, Ionian Sea) vs. Aegean-Black-Levantine seas	*Φ*_SC_ = 0.043 * *Φ*_ST_ = 0.044 * *Φ*_CT_ = 0.000 ns	_FSC_ = 0.046 * _FST_ = 0.042 ** _FCT_ = − 0.004 ns

*: Significant difference at *P* < 0.05; **: Significant difference at *P* < 0.01; ns: non-significant difference (*P* > 0.05)

### Evolutionary history and historical biogeography

BEAST analysis allowed unravelling a Cox1 subtitution rate of 4.87% per million years (Myr) with a 95% high posterior density interval (HPD) of 3.83–5.89%, which is within the range of substitution rates of 1.11–6.58% per Myr, estimated so far for the Cox1 gene in crustaceans [15, 47–50].

The yielded pattern of Cox1 haplotypes diversification clearly hints at the separation between two lineages or haplogroups (Fig. 3). Bayesian analyses of Cox1 sequences (posterior probability [PP] = 1.00) confirmed the mtDNA monophyly of *E. verrucosa*. The first mitochondrial clade (lineage 1) was found to be statistically supported (PP = 0.65); while the more genetically diversified clade (lineage 2) was poorly resolved (PP = 0.42) (Fig. 3). Within this latter clade, some derived nodes were shown to seemingly pre-date their ancestral nodes despite the strong convergence of the data. Referring to our earlier Bayesian phylogenetic reconstructions for intraspecific data [4, 8], we assume that such pattern could stem from the high frequency of diversification in relatively short period of time followed by longer period of relative stasis. When calibrating the haplotype phylogeny with the already determined Cox1 mutation rate, divergence among both retrieved lineages of *E. verrucosa* was found to occur approximately 66,270 years before present (YBP) (95% HPD: 37,100–114,194 YBP). Subsequent diversification within both retrieved mitochondrial lineages started nearly at the same time (lineage 2: 52,338 YBP [95% HPD: 31,719–78,900 YBP]; lineage 1: 50,066 YBP [95% HPD: 29,272–75,223 YBP]; Fig. 3).

Reconstruction of possible past geographic distributions of Cox1 haplotypes (Fig. 4) suggests that the origin of *E. verrucosa* was in a wide ancestral region encompassing the East Atlantic and Mediterranean regions (biogeographic region AB). The S-DIVA analysis allowed detecting a marked vicariance signal at the most ancestral node (basal node 59; Fig. 4) between the East Atlantic (regarded as the most likely ancestral range for lineage 1; biogeographic region A at node 38) and the Mediterranean Sea (considered a favored ancestral range for lineage 2; biogeographic region B at node 58). Re-analysis of the historical biogeography of *E. verrucosa* based on a total dataset of 143 sequences (excluding the GenBank sequences) allowed detecting the same patterns at the three most ancestral nodes (Additional file 5: Figure S1). Within lineage 1, several dispersal events were recorded at both the basal node (node 38) and the derived ones (nodes 32, 33, 35, 36, and 37) (Fig. 4). Within lineage 2, distinct dispersal waves (recorded at nodes 40, 42, 46, 49, and 50) were occasionally followed by vicariance events (noticed at nodes 39 and 45) (Fig. 4). The retrieved dispersal pathways within the two lineages suggest not only the occurrence of bidirectional dispersal events between the East Atlantic and Mediterranean Sea, but also further dispersal within the same region (Fig. 4). However, such interpretation should be cautiously considered owing to the small sample size of the East Atlantic in relation to the Mediterranean.

### Demographic history reconstruction

The analysis of the Tajima’s *D*, Fu’s *F*_S_, and Ramos-Onsins and Rozas’s R_2_ tests in the defined populations of *E. verrucosa* showed significant deviations from mutation-drift equilibrium for eight populations (Table 2). With the *R*_2_ test being more reliable to infer past demographic fluctuations in small sample size, the occurrence of a potential expansion event can be roughly highlighted for six populations of the warty crab (Table 2). The significant output of the three examined neutrality tests, together with the small and non-significant value of Harpending’s raggedness index *rg*, consolidate the scenario of a sudden demographic expansion for the whole dataset as well as for the Mediterranean Sea and its Western and Eastern basins (Table 2). A signature of past demographic expansion was also highlighted for the East Atlantic, mainly inferred from the significant Fu’s *F*_*S*_ along with the non-significant demographic index (*rg*) (Table 2).

Both demographic and spatial expansion models were accepted for the total dataset as well as for the East Atlantic and Mediterranean regions (Table 6). Only, the null hypothesis of expectation under a sudden demographic expansion model has not been statistically supported for the East Atlantic (Table 6). Both expansion scenarios were also validated for the Western and Eastern Mediterranean basins. Noteworthy, time since expansion, measured in mutational time units, allowed inferring marked older demographic and spatial expansion events recorded for the western Mediterranean than those retrieved for the eastern Mediterranean (Table 6).

**Table 6:** Test of both demograhic and spatial expansion models for the examined regions (and sub-regions) as well as for the whole dataset of *Eriphia verrucosa*

Examined region (and *sub-region*)/Tested expansion model	Demographic expansion			Spatial expansion		
	SSD	*P*	*τ*	SSD	*P*	*τ*
Atlantic Ocean	0.073	0.011	0.9	0.005	0.326	0.9
Mediterranean Sea	0.006	0.597	0.7	0.006	0.438	0.4
*western Mediterranean*	0.018	0.479	3.2	0.020	0.375	1.4
*eastern Mediterranean*	0.002	0.783	1.5	0.001	0.631	0.6
Whole dataset	0.006	0.556	1.0	0.006	0.344	0.5

*SSD* sum of squared deviations between observed and expected distributions under the tested expansion model. The probability of obtaining a simulated SSD greater than or equal to the expected was computed by 1000 random permutations. If this probability (*P*) was > 0.05, the expansion model is accepted. *τ* Time since expansion measured in mutational time units

**Fig. 5:**
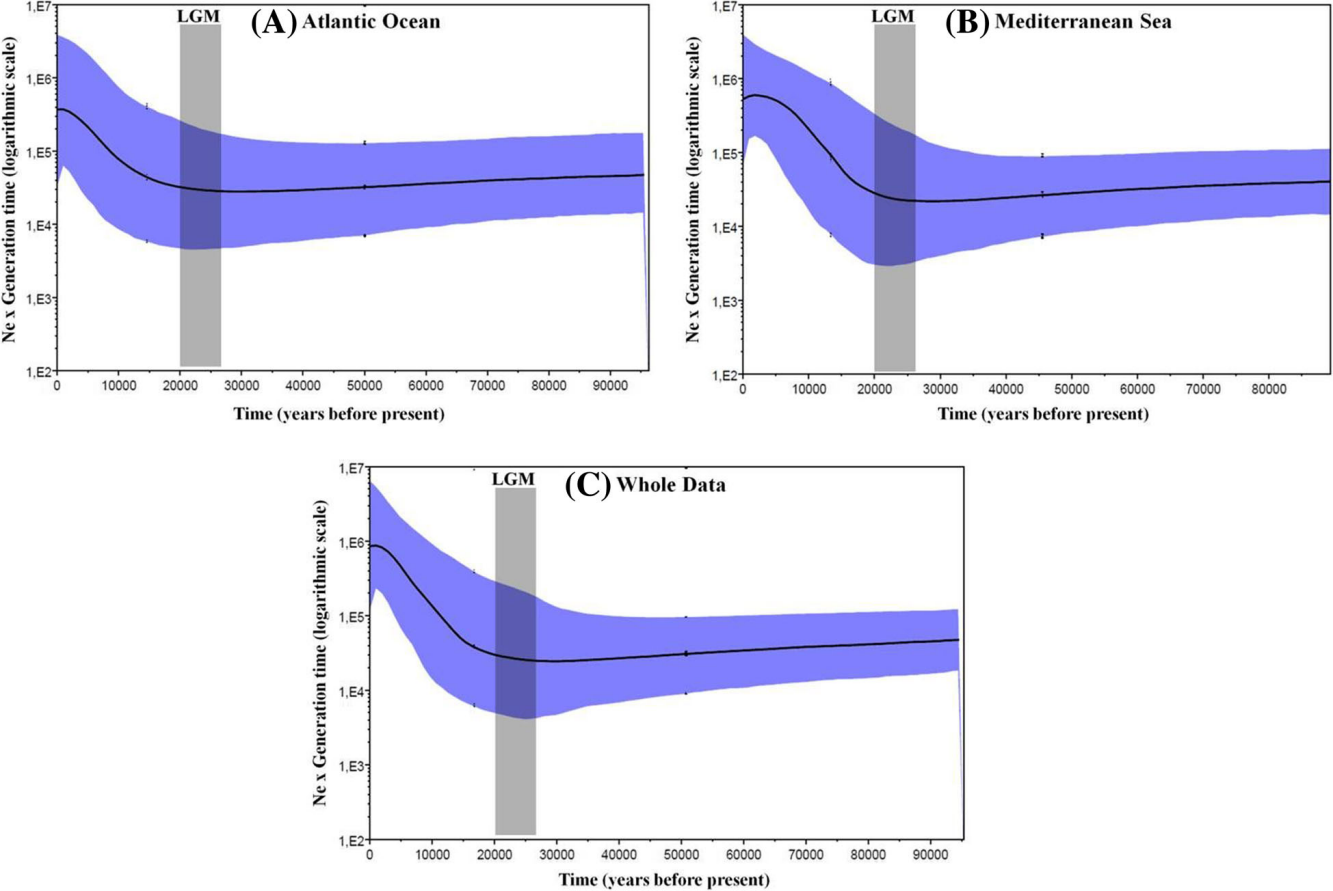
Bayesian skyline plots (BSP) for specimens of *Eriphia verrucosa* corresponding to the Atlantic Ocean (**a**), Mediterranean Sea (**b**) as well as to the whole dataset (**c**), showing changes in effective population size (Ne multiplied per generation time) over time (measured in years before present). The thick solid line depicts the median estimate, and the margins of the blue area represent the highest 95% posterior density intervals. The enclosed grey-shaded box corresponds to the Last Glacial Maximum (LGM) priod (between 26,500–20,000 years before present; [51])

The generated BSP plots, for the total dataset of *E. verrucosa* as well as for the East Atlantic and Mediterranean regions, showed a recent and sudden increase in effective population size, following a long stationary period (Fig. 5). The sudden expansion event occurred roughly at about 16,500 years ago (CI: 11,000–30,500 years ago) for the whole dataset (Fig. 5c). At the regional scale, East Atlantic specimens of *E. verrucosa* started expanding approximately at about 17,000 years ago (CI: 13,500–26,600 years ago) (Fig. 5a); while their Mediterranean counterparts underwent a marked significant increase in their effective size around 17,500 years ago (CI: 12,500–29,000 years ago) (Fig. 5b). All underlined expansion events markedly followed the Last Glacial Maximum period (between 26,500–20,000 years before present; [51]).

## Discussion

The present investigation is the first report on mitochondrial phylogeography and evolutionary history of *Eriphia verrucosa* across its entire distribution range. The main results of the study unveiled a lack of population genetic structure across biogeographic boundaries within the East Atlantic and Mediterranean, with noticeable genetic distinctiveness of the peripheral population from the Azores. They also showed that the late Pleistocene climate oscillations might have had a significant impact on shaping the genetic variability and structure of the examined warty crab species.

### Lack of phylogeographic structure across postulated barriers to gene flow

The outcomes of various population genetic analyses concur in unveiling lack of phylogeograhic structure across postulated barriers to gene flow. Such finding clearly contrasts with previous ones from other phylogeographic investigations of marine species [2, 8, 19, 21, 22, 32] and suggests that the biogeographic boundaries encompassing the surveyed region do not seem to restrict gene flow within *E. verrucosa*. Given its supposedly high larval dispersal potential [44] and continuous distribution across the intertidal belt of the East Atlantic and Mediterranean, we hypothesize that the observed pattern of genetic homogeneity could be attributed to the fact that long drifting larvae of the warty crab can reach very distant locations along the East Atlantic-Mediterranean littoral and assure genetic connectivity owing to the unidirectional surface current, called the Atlantic Current, originating from the East Atlantic, moving eastwards along the western Mediterranean coast and reaching into the eastern Mediterranean [52]. This assumption could be supported by the outcome of the TCS Cox1 genealogy, in which a wide distribution of the common haplotype H2 (Fig. 2), being shared by all populations, can be noticed.

Nevertheless, given that highly dispersive intertidal species, occurring in sympatry with *E. verrucosa*, have been shown to display a significant differentiation across boundaries [2, 4, 8, 15, 20, 21], we can assume that other factors (including larval behaviour and wide tolerance toward gradual shift in abiotic features) could be interacting with the high larval dispersal potential of the species and account for such recorded genetic homogeneity. In this context, we advance the hypothesis that widely tolerant larvae of *E. verrucosa* towards disparate environmental features of the Atlantic and the Mediterranean (i.e., salinity and temperature [53]) could overcome the wide variety of physical, chemical and biological processes that could potentially affect the probability of their transition from one stage to another [54, 55]. This would deprive the occurrence of local genetic structuring, potentially triggered by the effect of natural selection. Further experimental investigations on eco-biological properties of the warty crab larvae are required to test this hypothesis.

Alternatively, we can propose that high gene flow and a particular larval behaviour may not be the only causes of the lack of phylogeographic structure discerned within *E. verrucosa*, as other factors such as large effective population size could also be involved [5]. Indeed, given the high fecundity potential in this decapod species [41, 42], it is highly likely that the impact of genetic drift may be counteracted by large populations of the warty crab. Hence, the lack of population genetic structure over the surveyed distribution range could potentially stem from recent spatial and demographic expansions, as clearly observed in our study. In that case it may not necessarily reflect high levels of contemporary gene flow in this species, potentially induced by high larval dispersal potential and homogenizing oceanographic features [56].

While the above mentioned scenarios may provide possible explanations to the high genetic connectivity within *E. verrucosa*, they have to be considered with caution as the increase in sample size may yield new insights into the phylogeographic structure of the species across the postulated biogeographic boundaries. The recent study by Fratini et al. [21] showed that exhaustive increase in sample size allowed unraveling population genetic structuring (with mtDNA) in the marbled crab *P. marmoratus*, previously regarded as panmictic due to geographically and numerically limited datasets.

### Genetic differentiation of the Azores population

Opposed to a background of high regional and and sub-regional genetic connectivity, the East Atlantic peripheral population of Azores was shown to be genetically distinct. Such finding has been already reported for other marine invertebrate and vertebrate species with high dispersal potential [57–59]. This pattern of differentiation may hint at the crucial role played by contemporary isolating factors and/or historical processes in shaping this noticeable genetic isolation. Owing to the marginal position of the Azores Islands, we hypothesize that the impact of the countercurrents, roaming the Azores [60, 61], might render larval dispersal to more eastern locations difficult. This could prevent gene flow and account for the observed pattern of genetic differentiation. Alternatively, the recorded genetic distinctiveness of the Azores population could be attributed to the impact of historical processes linked to the palaeogeographic and palaeoclimatic evolution of the surveyed region. Given the particular genetic polymorphism (high haplotype diversity) recorded at these isolated islands at the westernmost edge of the species range, we rather suggest that this region might have served as a historical repository for genetic diversity during glacial low sea levels of the Pleistocene [62]. The impact of these historical events might have been maintained and intensified by the effect of particular oceanographic circulation, leading to the genetic differentiation of the Azores, as described in our study.

### Evolutionary history

In addition to the likely involvement of the above-discussed contemporary factors in shaping population genetic structure of *E. verrucosa*, historical processes might also have exerted significant impact on geographic distribution of genetic variability. This assumption is likely supported by the outcomes of regional genetic diversity, spatio-temporal patterns of genetic diversification, and demographic history. Thereby, a complex series of micro-evolutionary forces encompassing allopatric differentiation, followed by range expansion and secondary contact and admixture among refugial lineages, might have contributed to shaping genetic diversity and structure in this decapod species. Hence, we discuss the evolutionary history of *E. verrucosa* in the context of the following issues:

#### Potential glacial refugia within the East Atlantic and western Mediterranean

As far as can be inferred from the outcome of this study, several lines of evidence support the existence of potential marine glacial refugia in the East Atlantic and western Mediterranean. First, assessment and comparison of regional genetic diversification showed that East Atlantic and western Mediterranean samples of *E. verrucosa* were significantly more diversified than their eastern Mediterranean counterparts for most of the examined parameters. Second, BEAST mitochondrial genealogy highlights potential genetic signatures of late Pleistocene genetic divergence between two lineages. Third, reconstruction of possible past geographic distributions of Cox1 haplotypes, using the S-DIVA method, identified the East Atlantic and the Mediterranean Sea as the most likely ancestral ranges for both retrieved lineages within *E. verrucosa*. Such integrative evidences suggest that suitable habitats within the East Atlantic and western Mediterranean (although in need to be consolidated and specified by further palaeoenvironmental and palaeogeographic evidences) might have been important marine biodiversity refugia to retain ancestral relict populations of *E. verrucosa* during Pleistocene climate changes. This assumption is likely supported by the outcome of recent phylogeographic studies on other Atlanto-Mediterranean marine species, which have also identified potential glacial refugia within the East Atlantic (namely across the Iberian Peninsula and Macaronesia) and the western Mediterranean [14, 17, 30, 63–65]. However, based on the analysis of only single mtDNA marker in limited sample dataset, this finding has to be considered as tentative and need to be confirmed by the analysis of another variable nuclear marker along with numerically and geographically extended datasets. Furthermore, detailed investigation, involving fine-tuned ecological niche modeling, is required to specify the exact locations of the postulated refugia.

#### Late Pleistocene vicariance across the Gibraltar Srait followed by postglacial expansion and admixture among refugial lineages

Integrative outcomes of BEAST and RASP analyses allowed retrieving genetic imprints of a late Pleistocene vicariant event across the Gibraltar Strait, which occurred approximately around 66,300 years before present. This historical event corresponds to the glacial period of the late Pleistocene (Würm glaciation: 110,000–12,000 years before present; [66]). It is known that cyclic shifts in sea levels during the Pleistocene glacial and interglacial periods could have potentially affected palaeogeography as well as palaeoenvironment of the East Atlantic-Mediterranean region [28, 29]. Specifically, glacial cycles of the Pleistocene might have provided the opportunity for allopatric differentiation in intertidal marine invertebrate species with planktonic larval dispersal, not only due to potential loss of habitat and subsequent genetic drift, but also following the restriction of connectivity between the East Atlantic and Mediterranean across the Gibraltar Strait. Accordingly, we hypothesize that the two Cox1 lineages might have differentiated in distinct glacial refugia during the harsh climate of the Würm glacial cycle. During the Pleistocene glacial periods, marine regression (dropping in sea level of about 150 m) might have not only narrowed the Gibraltar Strait following the emergence of set of islands [67], but also led to the restriction of marine circulation between the East Atlantic and Mediterranean and subsequent interruption of gene flow across the Gibraltar Strait [19].

Contrary to what has been found for other Atlanto-Mediterranean decapod species [2, 16, 68], genetic discontinuity triggered by this historical isolation within *E. verrucosa* has not been maintained until the present, as gene flow has been intensively restored between allopatric populations. This pattern is clearly evidenced in the TCS mitochondrial genealogy and can be mainly explained by the outcome of demographic history reconstruction. In particular, the BSP analysis showed that all estimated expansion events for the East Atlantic and Mediterranean regions markedly followed the Last Glacial Maximum period (LGM, between 26,500–20,000 years before present; [51]). It has been postulated that the warming of the East Atlantic and Mediterranean Sea following the LGM might have led to the onset of favourable environmental conditions for the rapid growth and expansion of marine populations [17]. The simultaneous occurrence of demographic expansion of both regional groups supports this assumption and indicates that Atlanto-Mediterranean specimens of *E. verrucosa* might have responded similarly to the climatic change and new abiotic conditions. Hence, it is more likely that the evidenced postglacial demographic and range expansions for this species might have promoted secondary contact and admixture among potential refugial lineages.

#### Recent colonization of the eastern Mediterranean

Analysis of regional genetic diversification revealed significantly reduced levels of genetic variability in eastern Mediterranean specimens of *E. verrucosa*. This pattern was shown for all examined diversity parameters except for haplotypic richness. Based on these insights, and referring to the markedly younger demographic and spatial expansion events recorded for the eastern Mediterranean than those retrieved for the western Mediterranean, we hypothesize that areas located to the east of the distribution range of the species might have been recently and rapidly colonized from the nearest refugia, when new habitats became available again.

However, given the fact that the eastern Mediterranean was shown to be genetically more diversified than the western Mediterranean based on haplotypic richness, the validity of this assumption can be seriously questioned. Two possible explanations can be advanced in order to clarify this discrepancy. First, it has been shown that genetic diversity in re-colonized areas can be either higher or lower depending on the estimator [69]. Second, given the significantly lowest levels of private haplotypes recorded in the eastern Mediterranean against a background of higher haplotypic richness (after a rarefaction procedure), we can assume that unexpected increase in the latter parameter could be likely due to the fact that the eastern Mediterranean may correspond to a contact zone between founding haplotypes from potential refugial zones [62, 70]. According to the latter explanation, we hypothesize that the eastern Mediterranean might have been recently colonized from putative refugia within both the western Mediterranean and the East Atlantic. Nevertheless, it should be mentioned that owing to the limited examined samples in this study, these insights have to considered as tentative and need to be confirmed by analysis of another nuclear marker in exhaustive numerically and geographically datasets.

#### Evolutionary history scenario for *E. verrucosa*

Overall, based on the obtained preliminary results, we propose the following evolutionary history scenario for *E. verrucosa*: The harsh climatic changes, mainly mediated by significant climate cooling and dropping of sea levels, during the Würm glaciations, might have led to the restriction of biotic exchange across the Gibraltar Strait and consequently to the retreatment of specimens of the warty crab to specific refugia within the East Atlantic and Mediterranean Sea. Subsequent postglacial recolonization of suitable habitats, following the rise of sea level and amelioration of climate conditions, might have led not only to the resumed connectivity across the Gibraltar Strait but also to the recent colonization of the eastern Mediterranean. The resulting gene flow restoration among historically isolated populations across the Gibraltar Strait (which might have not had enough time to accumulate local significant genetic differences as well as adaptations to the disparate environmental conditions of the East Atlantic and the Mediterranean Sea) is still likely maintained by the impact of contemporary factors including oceanographic features, larval behaviour, and large effective population size.

## Conclusions

The results of the present study provide new insights into the phylogeography and evolutionary history of a common but poorly studied Atlanto-Mediterranean decapod species. Specifically, they contribute to the understanding of the impact of historical processes on shaping contemporary population genetic structure and diversity in intertidal marine species. Increase in the sample size and investigation of highly variable nuclear markers are highly required and recommended in future investigation in order to confirm the discerned pattern of phylogeographic structure and provide a more complete and nuanced view of the evolutionary history of this important species.

## Methods

### Sampling scheme and genomic DNA isolation

A total of 155 samples of *Eriphia verrucosa* were obtained from 35 locations across the whole distribution range stretching from the East Atlantic to the Black Sea (Table 1 and Fig. 1). Apart from incorporating newly collected material (143 specimens from 31 locations), all available Cox1 sequences in GenBank (12 sequences previously obtained from 4 locations) have been also included in the study in order to maximize delineation of phylogeographic patterns and reliably infer the evolutionary history of the examined decapod species. Details on the sampled locations, and the number of individuals analyzed per population, are reported in Fig. 1 and Table 1. In the field, a single pereiopod was removed from the sampled crabs and stored in absolute ethanol until genetic analysis (these appendages are re-gained in the successive moults). Subsequently, the animals were released in their original environments. Back to the laboratory, total genomic DNA was extracted from pereiopod muscle tissue by means of the Puregene kit (Gentra Systems: Minneapolis, MN55447, USA).

### Amplification and sequencing of mitochondrial Cox1 gene

Amplification of the mitochondrial Cox1 gene was carried out with the specifically constructed decapod primers COL6a and COH6 and/or COL6a and COH1b [71]. Details on the used mixture for PCR reactions as well as thermocycling conditions were cited in Deli et al. [15]. Visualization of PCR products (loaded on 1.5% agarose gel with GelRed staining) was carried out under UV light. Sequencing of the resulting Cox1 amplicons was carried out with the forward primer COL6a at LGC Genomics or Macrogen Europe. Inspection and edition of the obtained sequences were assured with Chromas Lite 2.1.1 [72]. After their alignment with Clustal W, implemented in BIOEDIT [73], Cox1 sequences were adjusted to a gene fragment of a 453 basepairs (bp) length for further statistical analyses. Sequences corresponding to the characterized Cox1 haplotypes were submitted to GenBank (accession numbers: MK493758-MK493787).

### Data and statistical analyses

#### Data analysis procedure

We tried to increase the sample size of the analyzed populations by combining collection sites featuring low number of specimens. In our pooling strategy, we decided to take into account two main criteria: (1) Assignment according to geographic origin of the specimens so that only geographically closed locations within the same region (East Atlantic or Mediterranean Sea) or sub-regions (western Mediterranean or eastern Mediterranean) can be combined. (2) Assignment considering the postulated barriers to gene flow (aimed to be investigated in this study for phylogeographic structure, see below). In the latter case, the pooling procedure was not applied on the geographically close locations lying across these biogeographic boundaries even if this pattern of combination may still lead to limited sample size as that obtained in the two defined populations of Northern Tunisia and Alboran Sea (Tables 1 and 2). Accordingly, samples from the 35 surveyed locations were combined into 15 populations (Table 2). It has to be mentioned that the adoption of this procedure in population genetic investigation may be problematic. It could seriously question the reliability of the outcome of the statistical analyses since any small change in some of the pooling decisions may change the significance of some statistical tests. However, in the absence of larger sample sizes that would surely avoid this rather inconvenient procedure, respecting the two above mentioned conditions for specimens pooling in our study would at least allow minimizing the drawback of the method. Moreover, the resulting initial genetic signals would be helpful for the build up to an in-depth further investigation and orientate the sampling focus if any specific issues need to be addressed.

#### Analysis of genetic variability and assessment of regional genetic diversification

The nucleotide compositions as well as the number of variable and parsimony-informative nucleotide sites were estimated with MEGA version 7.0.18 [74]. Measurements of DNA polymorphism, including numbers of haplotypes (*N*h) and polymorphic sites (*N*ps), haplotype (*h*) and nucleotide (*π*) diversities [75, 76], as well as mean number of nucleotide differences (K), were calculated for each population, each region (and sub-region), as well as for the total dataset by means of DnaSP version 5.10 [77].

Assessment and comparison of genetic diversification levels among the East Atlantic, western Mediterranean and eastern Mediterranean were carried out by the examination of seven genetic diversity indices (*h*, *π*, K, *Hap*r (haplotypic richness), *N*p (number of private haplotypes), *N*p/*N* (proportion of private haplotypes), and *N*p/*Hap*r (genetic endemism)). Owing to the difference among the sample sizes of the examined datasets, we applied a rarefaction procedure in order to discard the positive bias of sample size on inferred diversity. For each sample larger than the smallest one with the size *N* = 42, recorded for the East Atlantic region, a subsample of *N* individuals was drawn randomly and diversity indices were calculated. In order to infer reliable statistics, the procedure was repeated 30 times for each sample, and then the mean subsample diversity for each sample was calculated. In order to check for the consistency of the obtained results, and see whether the number of replicates could influence the outcome of comparison, analyses were re-carried out with 60 replicates. The unpaired *t*-test, as implemented in the online software T-Tests-Free Statistics and Forecasting Software (Calculators) version 1.2.1 [78], was used to assess inter-regional difference for each genetic diversity parameter.

#### Assessment of genealogical relationships among Cox1 haplotypes

Genealogical relationships among Cox1 haplotypes were examined and assessed through construction of the haplotype network with the statistical parsimony procedure included in the software TCS version 1.21 [79]. Correlation between genealogy and geographic distribution of Cox1 haplotypes was assessed by comparing the two measurements of population differentiation, *G*_ST_ (taking into account haplotype frequencies) and *N*_ST_ (considering relationship among haplotypes) [80, 81], in PERMUT & CPSRR version 2.0 [81].

#### Analyses of genetic differentiation and phylogeographic structure

Patterns of genetic differentiation within the total mitochondrial dataset (by means of one-level AMOVA [82]), and among pairs of populations, were assessed in ARLEQUIN version 3.1 [83]. Both nucleotide divergence (based on the Tajima-Nei model [84]) and haplotypic frequencies were used to infer these estimations. A total of 10,000 permutations were adopted to estimate significant genetic distances (*P* < 0.05). Accurate inference of the significance level was assured by the B-Y FDR correction [85] (based on the determined critical value of *P* < 0.00954).

The two-level AMOVA (as implemented in ARLEQUIN) was used to test hierarchical structuring of genetic variation within *E. verrucosa*, seeking for evidence of significant phylogeographic structure across postulated barriers to gene flow, such as the Gibraltar Strait, the Almería-Oran Oceanographic Front, the Siculo-Tunisian Stait, and the Peloponnese hydrographic break.

#### Evolutionary history and historical biogeography reconstruction

In order to reliably elucidate the evolutionary history of *E. verrucosa*, we specifically estimated the mutation rate of the Cox1 gene referring to the already surveyed phylogeny of the genus *Eriphia* by Lai et al. [86], whose findings suggest congruence between geography and phylogeny in *Eriphia*. We, therefore, implemented a calibration point derived from the biogeographic event of the Pleistocene glacial sea-level low stands, assuming that the vicariant speciation of Indian and Indo-West Pacific species of the genus *Eriphia* might have been triggered by the impact of dry exposure of the Sunda Shelf at the onset of the Pleistocene glaciation cycles which occurred approximately 2.588 million years ago (± 0.005) [87, 88]. The estimation of molecular clock rate was carried out in BEAST version 1.7.5 [89]. Before proceeding with the analysis, selection of the best-fit substitution model for the data was carried out with MODELTEST version 3.7 [90]. Cox1 sequences, corresponding to the eight examined species of the genus *Eriphia* by Lai et al. [86] (GenBank accession numbers: HM638034-HM638038; KC771023-KC771024; KC962408) as well as to the haplotypes retrieved in our study, were used in the analysis. The two parameter Birth-Death model (considered as an appropriate null model for species diversification [91]), along with an uncorrelated lognormal relaxed clock, were used as priors for the analysis. A total run of 30 million generations were designed for the Markov chain Monte Carlo (MCMC) simulations. The outcome of the Bayesian analysis was exhibited and checked in TRACER version 1.5 [92].

Temporal patterns of Cox1 haplotypes diversification were estimated in the software BEAST version 1.7.5 with a coalescent tree prior, suggested as an appropriate null model for intraspecific data [93]. A strict molecular clock along with the generalized time reversible (GTR) model of sequence evolution [94] (as determined by MODELTEST) were also chosen as priors to carry out the analysis. The diversification time of the examined Cox1 dataset was inferred after calibrating the retrieved haplotype phylogeny with the determined Cox1 gene mutation rate for *Eriphia*. The Bayesian analysis was accomplished after running the MCMC simulations for 30 million generations (sampled every 1000 generations), checking the generated outputs for convergence (Effective Sample Sizes, ESS, of all parameters > 200) in TRACER version 1.5, and summarizing the resultant trees in TreeAnnotator version 1.7.5 [89]. The obtained calibrated Cox1 genealogy was checked and shown in FigTree version 1.4.0 [95].

The biogeographic history of *E. verrucosa* was inferred from ancestral area reconstruction based on Cox1 haplotypes using the S-DIVA (Statistical Dispersal-Vicariance Analysis) method, as implemented in RASP version 3.2 [96]. S-DIVA is a parsimony method of historical biogeography [97] and is recommended if there is some a priori knowledge of vicariance events that could have played an important role in the biogeographic history of the examined taxon. Taking into account the geographic distribution of *E. verrucosa* Cox1 haplotypes, three main biogeographic regions were defined: (A) Atlantic Ocean, (B) Mediterranean Sea; and (C) Atlantic-Mediterranean. The Most Likely States (MLS) function, as implemented in RASP, was used to highlight the likelihood of possible ancestral range at each generated node of the phylogeny. The MCMC output and condensed tree exported from BEAST and TreeAnnotator, as well as the distribution of the Cox1 haplotypes through all defined biogeographical areas were loaded as ‘trees file’ and ‘distribution file’ respectively in order to perform S-DIVA analysis in RASP.

#### Inference of demographic history

Signatures of past demographic changes in the warty crab *E. verrucosa* were detected by the analysis of three neutrality tests (Tajima’s *D* [98], Fu’s *F*s [99], and Ramos-Onsins and Rozas’s *R*_2_ [100]). A total of 1000 coalescent simulations were employed in ARLEQUIN to estimate *D* and *F*s statistics, as well as in DnaSP to calculate the *R*_2_ index. Significantly negative outputs of *D* and *F*s are usually indicators of demographic expansion. In case of examined data with small size, the *R*_2_ test has more power and is more reliable than *D* and *F*s to detect population expansion. Evidence of sudden demographic expansion event was also checked via analysis of the Harpending’s raggedness index *rg* [101] in ARLEQUIN (with the significance of the test assessed with 10,000 replicates). All these measures were determined for each population, each region (and sub-region), as well as for the overall dataset.

Both demographic and spatial expansion [102] models, as implemented in ARLEQUIN, were assessed in the whole dataset as well as in the East Atlantic and Mediterranean Sea (including its Western and Eastern basins). The sum of squared deviations (SSD) between observed and expected distributions under the tested expansion model were used as a measure of fit, and the probability of obtaining a simulated SSD greater than or equal to the expected was computed by 1000 random permutations. If this probability was > 0.05, the expansion model was accepted. Time since expansion, measured in mutational time units (*τ*), was inferred for each study case.

Detailed information on the amplitude of historical demographic events that the warty crab populations could have undergone was also retrieved from the outcome of the Bayesian Skyline Plot (BSP) method [103]. BSP analyses were carried out, in BEAST version 1.7.5, for the East Atlantic and Mediterranean regions as well as for whole Cox1 dataset. A GTR substitution model and a strict molecular clock were set as priors for the analysis. Time since expansion was estimated with the specifically determined Cox1 gene mutation rate for *Eriphia*. Two MCMC simulations were run independently for 40 million iterations. After discarding the first 10% iterations (4 millions) as burn-in, LogCombiner version 1.7.5 [89] was used to combine the remaining outputs. BSP plots (corresponding to the three analyzed datasets) were generated in TRACER version 1.5 after checking for the data convergence.

## Additional files

Additional file 1: **Table S1.** Estimation of genetic diversity parameters in Atlantic and Mediterranean specimens of *Eriphia verrucosa*, based on two analyzed datasets (including and excluding retrieved sequences from GenBank). (DOCX 13 kb)

Additional file 2: **Table S2.** Pattern of assignment of the twelve Cox1 sequences of *Eriphia verrucosa* (retrieved from GenBank) to the detected haplotypes in this study. (DOCX 11 kb)

Additional file 3: **Table S3.** Geographic distribution of the 30 haplotypes of *E. verrucosa* recorded at the 15 defined populations within the East Atlantic Ocean and Mediterranean Sea. (DOCX 16 kb)

Additional file 4: **Table S4.** Analysis of pairwise genetic differentiation in a total dataset of 143 specimens of *Eriphia verrucosa* (excluding the twelve sequences from GenBank). (DOCX 17 kb)

Additional file 5. **Figure S1.** Analysis of the historical biogeography of *E. verrucosa* based on a total dataset of 143 sequences (excluding the GenBank sequences). (DOCX 304 kb)

## Abbreviations

°C: Degrees celsius
DNA: Deoxyribonucleic acid
mtDNA: Mitochondrial DNA
PCR: Polymerase chain reaction
UV: Ultra violet
