# Supplementary material for: Phylogeographic and evolutionary history analyses of the warty crab Eriphia verrucosa (Decapoda, Brachyura, Eriphiidae) unveil genetic imprints of a late Pleistocene vicariant event across the Gibraltar Strait, erased by postglacial expansion and admixture among refugial lineages
Source: BMC Evol Biol. 2019 May 17;19:105. doi: 10.1186/s12862-019-1423-2 (PMC6525375; doi:10.1186/s12862-019-1423-2)
Supplement: Supplementary file 1 — Table S1. Estimation of genetic diversity parameters in Atlantic and Mediterranean specimens of Eriphia verrucosa, based on two analyzed datasets (including and excluding retrieved sequences from GenBank). (DOCX 13 kb) [file 12862_2019_1423_MOESM1_ESM.docx]

**Table S1** Estimation of genetic diversity parameters in Atlantic and Mediterranean specimens of *Eriphia verrucosa*, based on two analyzed datasets (including and excluding retrieved sequences from GenBank). Values reported for each region as well as for the total dataset are: Sample size (*N*), number of haplotypes (*N*h), haplotype diversity (*h*), nucleotide diversity (*π*), and mean number of nucleotide differences (K)

| **Region** | ***N*** | ***N*h** | ***h*** | $\boldsymbol{\pi}$ | **K** |
| --- | --- | --- | --- | --- | --- |
| **Analyzed dataset including retrieved sequences from GenBank** | | | | | |
| **Atlantic Ocean** | 42 | 13 | 0.808 ± 0.052 | 0.0039 ± 0.0005 | 1.786 |
| **Mediterranean Sea** | 113 | 24 | 0.702 ± 0.046 | 0.0032 ± 0.0003 | 1.483 |
| **Total** | 155 | 30 | 0.733 ± 0.036 | 0.0034 ± 0.0002 | 1.575 |
| **Analyzed dataset without including retrieved sequences from GenBank** | | | | | |
| **Atlantic Ocean** | 33 | 9 | 0.733 ± 0.000 | 0.0037 ± 0.0000 | 1.663 |
| **Mediterranean Sea** | 110 | 22 | 0.686 ± 0.000 | 0.0031 ± 0.0000 | 1.404 |
| **Total** | 143 | 27 | 0.697 ± 0.000 | 0.0033 ± 0.0000 | 1.486 |
