# Supplementary material for: Phylogeographic and evolutionary history analyses of the warty crab Eriphia verrucosa (Decapoda, Brachyura, Eriphiidae) unveil genetic imprints of a late Pleistocene vicariant event across the Gibraltar Strait, erased by postglacial expansion and admixture among refugial lineages
Source: BMC Evol Biol. 2019 May 17;19:105. doi: 10.1186/s12862-019-1423-2 (PMC6525375; doi:10.1186/s12862-019-1423-2)
Supplement: Supplementary file 2 — Table S2. Pattern of assignment of the twelve Cox1 sequences of Eriphia verrucosa (retrieved from GenBank) to the detected haplotypes in this study. (DOCX 11 kb) [file 12862_2019_1423_MOESM2_ESM.docx]

**Table S2** Pattern of assignment of the twelve Cox1 sequences of *Eriphia verrucosa* (retrieved from GenBank) to the detected haplotypes in this study

| Population | N | Cox1 haplotype (number of sequences) |
| --- | --- | --- |
| Azores | 8 | H6 (1), H7 (2), H8 (3), H9 (1), H10 (1) |
| Cádiz | 1 | H2 (1) |
| Şile | 3 | H3 (2), H4 (1) |
