# Supplementary material for: Phylogeographic and evolutionary history analyses of the warty crab Eriphia verrucosa (Decapoda, Brachyura, Eriphiidae) unveil genetic imprints of a late Pleistocene vicariant event across the Gibraltar Strait, erased by postglacial expansion and admixture among refugial lineages
Source: BMC Evol Biol. 2019 May 17;19:105. doi: 10.1186/s12862-019-1423-2 (PMC6525375; doi:10.1186/s12862-019-1423-2)
Supplement: Supplementary file 4 — Table S4. Analysis of pairwise genetic differentiation in a total dataset of 143 specimens of Eriphia verrucosa (excluding the twelve sequences from GenBank). (DOCX 17 kb) [file 12862_2019_1423_MOESM4_ESM.docx]

**Table S4** Analysis of pairwise genetic differentiation in a total dataset of 143 specimens of *Eriphia verrucosa* (excluding the twelve sequences from GenBank), inferred from nucleotide divergence (*Φ*_ST_, below the diagonal) and haplotype frequency (*F*_ST,_ above the diagonal). Significant values in bold (*P* < 0.05) were calculated from 10,000 permutations. *: Significant values obtained after B-Y FDR correction

|  | Atlantic Ocean | | | Western Mediterranean | | | | | | Eastern Mediterranean | | | | | |
| --- | --- | --- | --- | --- | --- | --- | --- | --- | --- | --- | --- | --- | --- | --- | --- |
|  | AZO | CAI | SES | ALB | ALC | VAL | TAR | TYR | NTU | ETU | TRI | IST | ION | AG-B S | LEV |
| AZO | § | **0.252*** | **0.171** | 0.345 | **0.142** | **0.273** | **0.194** | **0.113** | 0.163 | **0.168** | **0.242** | **0.192*** | 0.080 | -0.006 | **0.242*** |
| CAI | **0.238*** | § | -0.038 | -0.024 | 0.025 | -0.082 | -0.006 | 0.076 | -0.061 | -0.040 | -0.048 | -0.017 | -0.022 | 0.078 | 0.002 |
| SES | 0.157 | 0.116 | § | 0.108 | -0.064 | -0.016 | -0.105 | -0.014 | -0.058 | -0.063 | 0.026 | -0.042 | -0.073 | 0.017 | 0.057 |
| ALB | 0.303 | -0.108 | 0.206 | § | 0.111 | -0.110 | 0.142 | 0.147 | 0.000 | 0.043 | -0.089 | -0.003 | 0.025 | 0.127 | -0.094 |
| ALC | **0.180** | -0.022 | 0.043 | -0.114 | § | 0.038 | -0.026 | -0.031 | -0.040 | -0.019 | 0.046 | -0.000 | -0.072 | 0.012 | 0.079 |
| VAL | **0.259** | -0.089 | 0.146 | -0.175 | -0.050 | § | 0.018 | 0.087 | -0.090 | -0.042 | -0.105 | -0.031 | -0.025 | 0.080 | -0.046 |
| TAR | **0.157** | 0.077 | -0.116 | 0.144 | 0.042 | 0.093 | § | 0.022 | 0.004 | -0.041 | 0.059 | 0.004 | -0.025 | 0.059 | 0.105 |
| TYR | **0.238*** | 0.001 | 0.086 | -0.114 | -0.031 | -0.033 | 0.082 | § | -0.023 | 0.009 | 0.072 | 0.017 | -0.030 | -0.025 | **0.110** |
| NTU | **0.316** | -0.011 | 0.212 | 0.000 | -0.049 | -0.043 | 0.172 | -0.050 | § | -0.115 | -0.111 | -0.083 | -0.106 | -0.043 | -0.081 |
| ETU | **0.218*** | -0.060 | 0.098 | -0.128 | -0.027 | -0.097 | 0.065 | -0.007 | -0.087 | § | -0.051 | -0.020 | -0.046 | 0.018 | 0.032 |
| TRI | **0.383*** | 0.008 | 0.327 | -0.166 | 0.036 | -0.051 | 0.247 | 0.036 | 0.017 | -0.035 | § | -0.019 | -0.022 | 0.051 | -0.049 |
| IST | **0.265*** | -0.025 | 0.149 | -0.134 | -0.014 | -0.061 | 0.118 | -0.005 | -0.031 | -0.023 | 0.018 | § | -0.040 | 0.011 | 0.004 |
| ION | **0.162** | -0.049 | 0.081 | -0.132 | -0.082 | -0.079 | 0.062 | -0.034 | -0.043 | -0.051 | 0.026 | -0.039 | § | -0.051 | -0.006 |
| AG-B S | **0.185** | -0.021 | 0.152 | -0.124 | -0.023 | -0.056 | 0.119 | -0.007 | -0.022 | -0.027 | 0.032 | -0.035 | -0.069 | § | 0.063 |
| LEV | **0.339*** | 0.048 | **0.316** | -0.174 | 0.058 | -0.005 | **0.261*** | 0.030 | -0.035 | 0.040 | -0.004 | 0.035 | 0.020 | 0.015 | § |

AZO: Azores; CAI: Canary Islands; SES: Sesimbra; ALB: Alboran Sea; ALC: Alicante; VAL: Valencia; TAR: Tarragona; TYR: Tyrrhenian Sea; NTU: Northern Tunisia; ETU: Eastern Tunisia; TRI: Tripoli; IST: Istra; ION: Ionian Sea; AG-B S: Aegean-Black seas: LEV: Levantine Sea.
