# Supplementary material for: Phylogeographic and evolutionary history analyses of the warty crab Eriphia verrucosa (Decapoda, Brachyura, Eriphiidae) unveil genetic imprints of a late Pleistocene vicariant event across the Gibraltar Strait, erased by postglacial expansion and admixture among refugial lineages
Source: BMC Evol Biol. 2019 May 17;19:105. doi: 10.1186/s12862-019-1423-2 (PMC6525375; doi:10.1186/s12862-019-1423-2)
Supplement: Supplementary file 5 — Figure S1. Analysis of the historical biogeography of E. verrucosa based on a total dataset of 143 sequences (excluding the GenBank sequences). (DOCX 304 kb) [file 12862_2019_1423_MOESM5_ESM.docx]

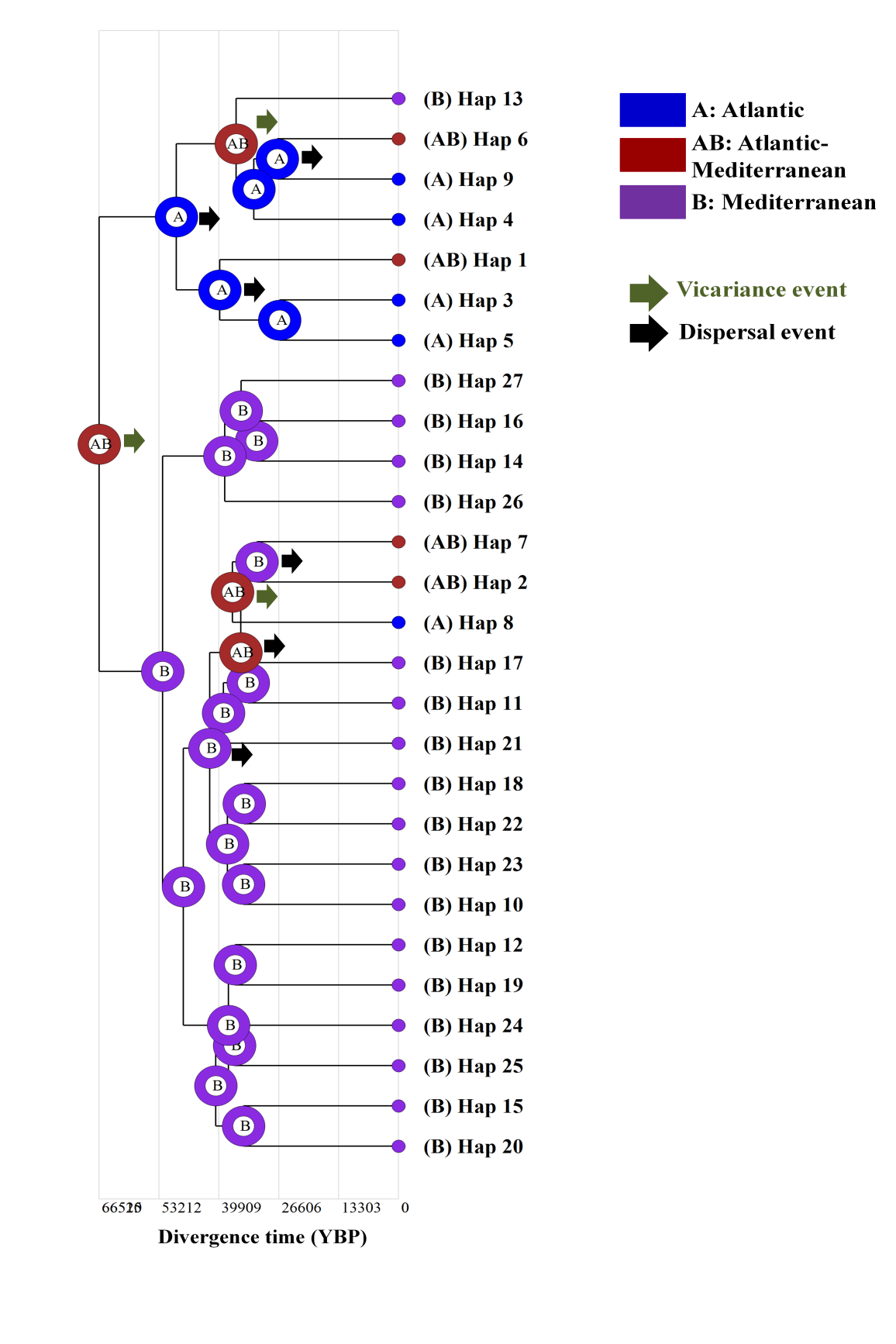


**Fig. S1** Analysis of the historical biogeography of *E. verrucosa* based on a total dataset of 143 sequences (excluding the GenBank sequences). Couloured pie charts on each node show the likelihood of occurrence of each ancestral haplotype at an inferred ancestral biogeographic region. Graphical results of ancestral distributions at each node of the phylogeny were obtained by S-DIVA method, as implemented in RASP version 3.2. Biogeographic regions are shown in different colors and denoted with alphabetic letters (A, B, and AB). Green arrows indicate vicariance events at the corresponding nodes; while black arrows highlight possible dispersal events
